# Barriers and Facilitators to Exercise in Older Adults Awaiting Kidney Transplantation and Their Care Partners

**DOI:** 10.1016/j.xkme.2023.100779

**Published:** 2023-12-13

**Authors:** Anoop Sheshadri, Jessica R. Elia, Gabriel Garcia, Gary Abrams, Deborah B. Adey, Jennifer C. Lai, Rebecca L. Sudore

**Affiliations:** 1 Division of Nephrology, Department of Medicine, University of California, San Francisco; 2 San Francisco Veterans Affairs Medical Center, San Francisco, California; 3 University of California Weill Institute for Neurosciences, San Francisco, California; 4 Division of Gastroenterology/Hepatology, Department of Medicine, University of California, San Francisco; 5 Division of Geriatrics, Department of Medicine, University of California, San Francisco, California

**Keywords:** Physical activity, exercise, dialysis, care partner, motivations, barriers, facilitators, pretransplant, qualitative

## Abstract

**Rationale & Objective:**

Despite guidelines calling to improve physical activity in older adults, and evidence suggesting that prekidney transplant physical function is highly associated with posttransplant outcomes, only a small percentage of older patients treated with dialysis are engaged in structured exercise. We sought to elucidate barriers and facilitators of exercise among older adults treated with dialysis awaiting transplant and their care partners.

**Study Design:**

Individual, in-depth, cognitive interviews were conducted separately for patients and care partners through secure web-conferencing.

**Setting & Participants:**

Twenty-three patients (≥50 years of age, treated with dialysis from the University of San Francisco kidney transplantation clinic, with a short physical performance battery of ≤10) and their care partners.

**Analytical Approach:**

All audio interviews were transcribed verbatim. Three investigators independently coded data and performed qualitative thematic content. The interview guide was updated iteratively based on the Capability Opportunity Motivation Behavior model.

**Results:**

Patients’ median age was 60 years (57 ± 63.5) and care partners’ median ages was 57 years (49.5 ± 65.5). Thirty-nine percent of patients and 78% of care partners were female, 39% of patients and 30% of care partners self-identified as African American, and 47% of dyads were spouse or partner relationships. Major themes for barriers to pretransplant exercise included lack of understanding of an appropriate regimen, physical impairments, dialysis schedules, and safety concerns. Major facilitators included having individualized or structured exercise programs, increasing social support for patients and care partners, and motivation to regain independence or functionality or to promote successful transplantation.

**Limitations:**

Participants geographically limited to Northern California.

**Conclusions:**

Although patients and care partners report numerous barriers to pretransplant exercise and activity, they also reported many facilitators. An individualized, structured, home-based exercise program could circumvent many of the reported barriers and allow older patients to improve pretransplant physical function.

## Plain-Language Summary

Although exercise can improve the fitness of older adults treated with dialysis for kidney transplantation and reduce posttransplant complications, many such individuals do not exercise. We sought to elicit perspectives on barriers and facilitators to prekidney transplant exercises from older adults treated with dialysis and their care partners. We found that although patients and care partners had unique perspectives, they shared many barriers (such as physical and/or cognitive impairment, difficulty scheduling around dialysis, lack of guidance on exercise, and reduced exercise motivation related to dialysis) and several facilitators (such as desire to regain functionality and participate in life and motivation for successful transplantation). A shared interest among patients and care partners in joint participation in structured and home-based exercise may represent a tool to overcome barriers to pretransplant exercise.

Kidney failure is considered a disease of premature aging,^1^, ^2^, ^3^ with patients considered physiologically older even by age 50.^4^, ^5^, ^6^, ^7^, ^8^ There are more than 600,000 older adults^9^ treated with dialysis in the United States, and therefore the number of older patients awaiting kidney transplantation is rapidly rising.^10^ Patients greater than 50 years and older represent nearly 70% of the waitlisted candidates,^11^ and the percentage of those aged ≥65 years has doubled in the last 2 decades to more than 20% of kidney transplant candidates.^12^ A successful kidney transplantation is associated with survival benefits in older patients compared with remaining receiving dialysis,^13^ and improvements in quality of life and functionality.^14^, ^15^, ^16^, ^17^, ^18^ However, older patients are at extremely high risk for functional impairment and mortality.^5^^,^^19^

Exercise interventions are known to improve physical function and quality of life outcomes in patients treated with dialysis,^20^, ^21^, ^22^, ^23^, ^24^ and observational studies have shown an association between physical activity and mortality.^25^^,^^26^ Targeted exercise interventions before transplantation have the potential to improve physical function and activity in waitlisted older patients and help them maximize their benefit from transplantation. Unfortunately, only a small percentage of patients treated with dialysis (who make up the majority of patients awaiting kidney transplantation)^11^ are able to participate in such interventions.^27^ Previous research suggests that may be due in part to dialysis-related and symptoms-related barriers such as fatigue and dyspnea,^28^, ^29^, ^30^ which may be compounded in older adults by more limited mobility,^5^ fear of falling,^31^ and polypharmacy.^32^ Many patients receiving dialysis also require support from care partners^33^ whose own quality of life may be impacted by their burden of care.^34^, ^35^, ^36^ Indeed in our previous study of a trial of a walking intervention for patients receiving dialysis irrespective of waitlist status,^37^ older patients identified unique barriers to walking participation, such as difficulty with transport or care partner concerns with walking safely.

To design exercise interventions that align with both patient and care partner preferences and abilities, it is essential to include the voices of older patients treated with dialysis awaiting kidney transplantation and their care partners.^38^^,^^39^ Therefore, we used qualitative research methods to explore barriers to and facilitators of an exercise program for older patients awaiting kidney transplantation from the perspectives of a diverse sample of both older patients and their care partners. We applied the Behavior Change Wheel (BCW) framework^40^, ^41^, ^42^, ^43^ and the Capability Opportunity Motivation Behavior (COM-B) model to map behavioral barriers and facilitators to intervention strategies for future interventions that serve the needs of both older patients and care partners.

## Methods

### Setting and Recruitment

We recruited patients and care partners who were previously enrolled in a study assessing physical performance among patients awaiting kidney transplantation at the University of San Francisco (UCSF) Connie Frank kidney transplant clinic. Participants were selected through the use of a purposive sampling technique to select a diverse group of patients with respect to age, biological sex, race or ethnicity, and range of waitlist times to elicit a broad range of perspectives.^44^ All potential participants were screened for eligibility by study coordinators. Patients were included if they were aged 50 years or older, currently treated with hemodialysis or peritoneal dialysis, awaiting either a living donor or deceased donor kidney transplantation, and had scored ≤10 on the short physical performance battery (SPPB), a marker of poor physical functioning associated with prefrailty and higher risk of major mobility disability. As part of the requirements for kidney transplantation at UCSF, potential transplant recipients are required to identify a primary care partner. After confirming eligibility of and obtaining consent from the patient, we then confirmed permission to consent the care partner as a requirement for entrance into the study. Consenting patient-care partner dyads were enrolled in the study. Participants were excluded if they were unable to speak English or if they were unable to obtain telephone or internet access to conduct remote interviews.

During telephone or in-person screening before interviews, we also collected participant age, biological sex, and race or ethnicity. For patients, we also collected data on dialysis prescriptions and time on the transplant waitlist. This study was approved by the institutional review board at the University of California, San Francisco (#19-29150).

### Procedures

Patient participants were contacted over the phone to determine their willingness to participate and to obtain permission to contact their primary care partner. Informed consent was obtained electronically. We conducted separate individual interviews of older patients treated with dialysis awaiting kidney transplantation and their care partners from September 2020 to April 2021, continuing recruitment until content saturation was achieved. Interviews were conducted by 2 facilitators with experience in dialysis and transplantation (AS, a male nephrologist and clinical researcher with formal qualitative research training, and GG, a male clinical research coordinator with informal qualitative research training) through secure web-conferencing (UCSF Zoom). Interviewers had no previous relationship with interviewees before study commencement. Participants were informed about the overall goals of the research project (eg, identifying barriers and facilitators to exercise before transplantation with the goal of understanding patient and caregiver perspectives). Interviews were audio-recorded and transcribed by an independent transcriptionist. To provide further context for the results, patients were also remotely administered the Center for Epidemiological Studies-Depression Scale^45^ and the Montreal Cognitive Assessment.^46^

### Interview Guide

A semistructured interview guide was prepared based on previous literature and input from experts in geriatrics, nephrology, and transplantation. The guide was updated using an iterative process based on the BCW framework^40^, ^41^, ^42^, ^43^ and piloted with transplantation providers and a patient-caregiver dyad (not included in this analysis). The BCW uses the Capability Opportunity Motivation Behavior (COM-B) model to understand targeted behaviors (eg, SPaRKT-2 engagement) in context. COM-B specifies that changing behavior requires changing individuals’ capability (psychological, physical, or cognitive), opportunity (social or physical), and motivation (reflective or automatic) regarding the behavior. BCW posits 9 intervention functions (eg, education, or environmental restructuring) and 7 categories of policy (eg, guidelines or service provision) that can guide intervention development.^47^ Thus, the COM-B model and BCW framework provide a basis for considering and translating stakeholder input into behavior change targets and intervention strategies needed to overcome barriers in a given context. Topics for both patients and care partners included general experience on the transplantation waitlist, overall quality of life, functional status and ability to perform activities (including caregiving), experience of symptoms, current exercise or activity status, motivators for exercise, barriers to exercise, rapport with care partner (or patient), communication with the medical and transplantation team, and input into potential programs for prehabilitation. Interviews lasted 60-90 minutes. Each patient-caregiver dyad was interviewed once.

### Data Analysis

All in-depth, cognitive interview data were audio-recorded, transcribed verbatim, and imported into ATLAS.ti^48^ software for qualitative analysis. To ensure rigor in data analysis, we developed a common code book (open coding) to document emerging concepts and overarching themes based on standardized interview guides. The preliminary coding scheme followed the interview guide and elements determined a priori from the literature and was refined through serial review of the transcripts.

Although reviewing data, research personnel noted recurring patterns using memoing (a process of recording reflective notes).^49^ After each interview, personnel (AS and GG) prepared a report of the top themes for each section of the interview. Through thematic content analysis of all transcripts,^50^, ^51^, ^52^ research personnel (AS, GG, and JE—a physical therapist with formal clinical research training and informal qualitative research training) identified “theme categories” to provide insight into specific barriers and facilitators to exercise interventions in older patients awaiting kidney transplantation and their care partners. The coding scheme was consistently refined using the constant comparative method.^53^ Overarching themes were identified, and disagreements were resolved by consensus. We ensured trustworthiness through clear inclusion and exclusion criteria, standard interview guides and coding schemes, and a clear audit trail for coding. In addition, we performed member-checking with participants before finalizing our codebook and received participant feedback on codes and themes. We evaluated trustworthiness through calculation of concordance of applied codes to the same segments of text, reaching an interrater reliability of 82%. Patients’ baseline characteristics were summarized as median (25th and 75th percentile) for continuous variables or frequency and percentage for categorical variables. We have also included ID numbers, with ID P1-P23 assigned to older patients and ID C1-C23 assigned to care partners.

## Results

### Participant Characteristics

We recruited 23 older patients and 23 care partners. The median age of patients was 60 (57 ± 63.5) and the median age of care partners was 57 (49.5 ± 65.5) (Table 1). Thirty-nine percent of patients and 78% of care partners were female. The largest racial group among both patient and care partner respondents was African American (39% and 30%, respectively), and 22% of both patients and care partners identified as being of Hispanic ethnicity. The most common patient-care partner relationship was spouse or partner (47%), followed by friend (26%). Among patients, 44% were treated with in-center hemodialysis, 52% with peritoneal dialysis, and 1 patient with home hemodialysis. The median waitlist time at the time of interview was 27.7 months (20.1-50.9), and 57% of patients were active on the waitlist at the time of interview.

**Table 1:** Participant Characteristics (N = 46)

Characteristics	Patients (n = 23)	Care Partners (n = 23)
**Age (y), mean ± SD**	60 (57 ± 63.5)	57 (49.5 ± 65.5)
**Female, n (%)**	9 (39%)	18 (78%)
**Race, n (%)**		
Asian	5 (22%)	5 (22%)
African American	9 (39%)	7 (30%)
White	4 (17%)	5 (22%)
Other	5 (22%)	6 (26%)
**Ethnicity, n (%)**		
Hispanic	5 (22%)	5 (22%)
**Dialysis modality, n (%)**		
In-center hemodialysis	10 (44%)	
Peritoneal dialysis	12 (52%)	
Home hemodialysis	1 (4%)	
**Waitlist time (mo), 25th-75th**	27.7 (20.1-50.9)	
**% Active on waitlist**	57%	
**BMI, n (mean ± SD)**	30.1 (26.1 ± 33.1)	
**SPPB (25th-75th)**	9 (8.5-10)	
**CES-D**	12 (9.8, 19)	
**MOCA**^a^	25 (21, 28)	
Unimpaired	9 (39%)	
Mild cognitive impairment	10 (43%)	
Moderate cognitive impairment	2 (2%)	
**Care partner relationship to patient**		
Spouse or partner		11 (47%)
Friend		6 (26%)
Parent		2 (9%)
Child		2 (9%)
Sibling		2 (9%)

Abbreviations: SPPB, short physical performance battery (0-12, with lower scores being worse and scores ≤10 associated with high risk of developing major mobility disability); CES-D, center for epidemiological studies-depression (scores ≥16 considered at risk for depression among hemodialysis patients); MOCA, Montreal cognitive assessment.

a 2 patients were unable to complete MOCA because of technical issues.

When asked about current exercise or intentional physical activity, 78% of patients and 57% of care partners reported consistent engagement, primarily in the form of walking (Table 2). Only 6 patient-care partner pairs currently exercised together. However, when asking care partners about their willingness to exercise or perform intentional activity with their patient before transplantation, 20 care partners (87%) reported they would be willing to do so if given a structured program and if given the opportunity.

**Table 2:** Patient and Care partner Exercise or Physical Activity Status and Counseling

	Patients (n = 23)	Care Partners (n = 23)
**Current exercise or physical activity status**		
At least some exercise or intentional physical activity∗	18 (78%)	13 (57%)
Walking	9	9
Stair-climbing	1	
Biking	4	2
Weights or body weight	7	3
Stretching	4	1
Yoga	2	
Physical therapy program	3	
Other		1
Exercises or performs intentional physical activity with care partner	6 (26%)	-
Not exercising or engaging in intentional physical activity	5 (22%)	4 (17.4%)
Not asked	0	4 (17.4%)
**Counseling on exercise or physical activity for patient**		
Received counseling to increase exercise or physical activity	6 (26%)	1 (4%)
Type:		
Walking	2 (33%)	1
No specifics	4 (67%)	
Source:		
Primary nephrologist	4	
Transplant team	1	
Other physician	1	1
Not counseled on exercise or activity for patient	17 (74%)	20 (87%)
Not asked	0	2

Most participants (74% of patients and 87% of care partners) had never been counseled on any type of exercise by a member of their care team. Of the 6 patients who did report being counseled, only 2 received any type of specific instruction (both related to walking only). The only care partner to report receiving counseling for the patient received information on walking.

**Figure 1:**
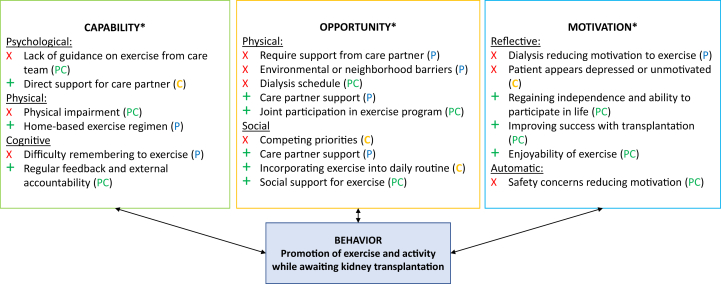
Barriers and facilitators to exercise among older patients awaiting transplantation and their care partners—the COM-B model. ^a^COM-B, capability: ability to enact the behavior, motivation: beliefs or emotions that active or inhibit behavior, opportunity: factors in the environmental or cultural milieu that influence behavior. Barriers are noted with an X. Facilitators are noted with a +. P, C, and PC are used for themes that are patient-specific (P), care partner-specific (C), or apply to both (PC).

**Figure 2:**
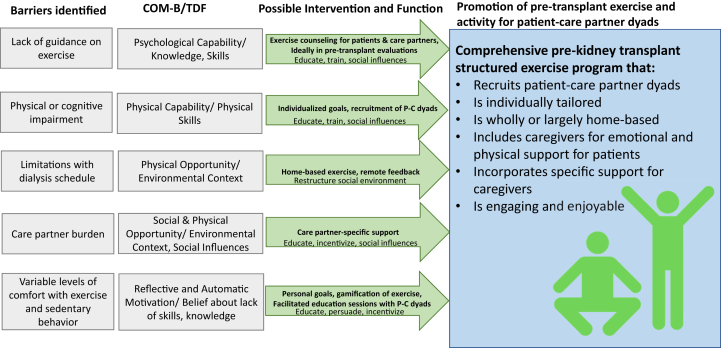
Evidence-based intervention strategies to overcome patient and care partner barriers to pretransplant exercise and activity. COM-B, capability: ability to enact the behavior, motivation: beliefs or emotions that active or inhibit behavior, opportunity: factors in the environmental or cultural milieu that influence behavior. TDF, theoretical domains framework; P-C, patient-care partner.

We identified pertinent barriers and facilitators to pretransplant exercise and activity from the perspective of older patients (Tables 3 and 4) and care partners (Tables 5 and 6). Fig 1 synthesizes our thematic analysis of pertinent barriers and facilitators to increasing exercise for the patient before transplantation from the perspective of both patients and care partners. Potential behavioral interventions to address barriers or leverage facilitators are listed in Fig 2.

**Table 3:** Patient Barriers to Pretransplant Exercise and Activity

*COM-B*	Theme	Quotation
**Capability**	Lack of information on appropriate exercise regimen	“…my kidney doctors have always encouraged me to exercise. But in terms of being more specific of how much I should exercise per day…that never happened.” (ID P1)
	Physical or Cognitive Impairment	“I couldn’t even walk to [my neighbor’s] house. I had to drive our car, just 2 doors down. I mean, it was that bad.” (ID P1) “Dialysis for sure is kind of a domino effect… So right now, I feel very aged, very aged.” (ID P20)
**Opportunity**	Dialysis schedule makes exercise difficult	“You know, there's nothing else I can do…I got to do dialysis every 4 hours, you know?” (ID P4) “I didn’t do well in [physical] therapy because I was so weak [with dialysis].” (ID P13)
	Require support from care partner to be able to exercise	“[My care partner] loads me on the bus…They have to take me [where I need to go] and when I’m done, they bring me home and then they have to be here to let me in.” (ID P13) “If…I have to tell myself what to do, maybe I'll do it, maybe I won’t.” (ID P20)
	Environmental or neighborhood-related barriers	“First… it got really cold, and then when it warmed up we started the [wildfires], and it wasn't healthy to be outside.” (ID P5) “Walking up that hill, it's kind of hard, you know? Takes all the wind out of me.” (ID P11)
**Motivation**	Reduced motivation to exercise	“[Dialysis] just takes you out of your mood or whatever you had planned that you were going to do that day and you get frustrated.” (ID P10) “I couldn’t walk [up a hill], and I have never had that experience in my whole entire life. I think I passed out twice and…[so] I haven’t got to the push-ups yet.” [ID P12]

**Table 4:** Patient Facilitators to Pretransplant Exercise and Activity

*COM-B*	Theme	Quote
**Capability**	Individualized home exercise regimen	“I wouldn’t mind a home-based [exercise program] because you can keep to that no matter what …whereas the gym, you’re relying on a lot of different other things and I don’t feel safe…” (ID P10) “…you have to have a [structured] program for it. It’s not just, ‘Oh, okay…I [just] have to work harder now…’” (ID P2)
	Accountability for goals with regular feedback	“If I had a coach and if I had someone to work with…there's something I can achieve. Versus doing it myself, it has been really difficult.” (ID P12) “The structured coaching for me is kind of an examination…You want to do well.” (ID P16) “I’m also in control…as long as I have a tool which measures what I’m achieving.” (ID P8)
**Opportunity**	Care partner support in exercise	“(Care partner) support would help motivate me.” (ID P10) “…when we go for walks…I can hold onto [my care partner] and push myself further.” (ID P3)
**Motivation**	Regaining functionality and ability to participate in life	“…I’m doing it purely to stay fit, to be able to be mobile… I think to be an active participant in your own health is very, very important.” (ID P8) “I used to use a walker before and then I switched to a cane…I want to be walking by myself.” (ID P14) “I want to do anything as much as I was doing before. Like exercise to motivate myself” (ID P14)
	Improving success with transplantation	“I wanted to have a better chance… the healthier I am going into it, the healthier I’ll be getting out of it. My partner [said] ‘You have to start now if you’re going to make a change.’” (ID P16) “People have -- more longevity if they are physically active. So, that kind of, you know, stressed me a little bit, because I thought, oh my gosh -- you should start, even before [transplantation].” (ID P20)
	Social engagement as a motivator	“When I met [another patient] and I saw how active she was I thought ‘Wow, so it's possible,’ so she encouraged me, and I hope that I could be an encouragement to others.” (ID P5) “I mean, when you see other people exercising, it kind of motivates you too because sometimes you see people in worse shape than you… So, you say ‘Well, if they’re doing it…’” (ID P7)

**Table 5:** Care partner Barriers to Promotion of Pretransplant Exercise and Activity for Patients

*COM-B*	Theme	Quote
**Capability**	Lack of guidance from patient’s care team	“I didn’t remember them saying, ‘Well, it’s really important you keep up your exercises and eat well,’ or whatever. They weren’t real specific.” (ID C2) “[On lack of support] I don’t feel personal assistance [from] a doctor telling me ‘…We’re going to try this and you’ll feel better.’” (ID C13) “They could [explain to] us the caregiving aspect… what do we look out for?” (ID C14)
	Concerns with care partner’s own health	“[On burden of care]: It’s continuous. Blood pressure goes up. Heart rate goes up…it’s taking its toll on me.” (ID C13) “… we’re a little bit more cautious. We know that if we fall, our bones will be a little bit more brittle.” (ID C15)
**Opportunity**	Competing priorities or lack of time	“I have a lot of responsibilities to take care of… I’m kind of the go-to person in my family, extended family, and so I get dragged into a lot of stuff for mostly medical issues.”(ID C8)
	Dialysis schedule limiting ability to exercise	“…he [sequesters] himself for… 12, 16 hours…? And maybe he wakes up in the morning… and the cycle hasn't completed, so he’s stuck in bed for another however many hours.” (ID C21) “…on dialysis days he comes home and he’s…wiped out -- functionally just exhausted.” (ID C23)
**Motivation**	Patient does not appear motivated or appears depressed	“He’s telling me that he can't walk that far. His back starts hurting. So I just stopped asking” (ID C4) “The years grind on, and all the plans that he had…they have to basically die, right?… I can only imagine how it grinds on you.” (ID C21)
	Concerns about patient safety or ability	“He wanted to be able to walk [with his friend]… and I said, ‘Well, I would be concerned that he would trip.’” (ID C2) “He likes to work… but -- sometimes I got to tell him, ‘You can't be doing that.’” (ID C18)

**Table 6:** Care Partner Facilitators to Promotion of Pretransplant Exercise and Activity for Patients

*COM-B*	Theme	Quote
Capability	Direct support for care partner	“I had a doctor taught me how to take time for myself, so I kind of get up like a half an hour early in the morning and that’s my own just-me time.” (ID C10)
Opportunity	Joint participation in individualized exercise program	“I like the idea of somebody monitoring…[being] accountable to someone other than myself.” (ID C6) “Instead of just throwing him into water, I should be in the water with him and show him ‘this is how you float.’” (ID C15) “If someone told her what her what needs to be met then…I would do it too…we’ll work out or go walking more or whatever the case is.” (ID C17)
	Incorporating into daily routine	“If I can’t make it to the banks, I make him walk so he can get the walking exercise.” (ID C14) “[His dietitian] recommended lifting weights. So…I said ‘I need detergent, please’… and he’ll go and get [the heavy bottle]. You can’t just [say] ‘Go lift some weights.’ They’re not going to do it.” (ID C15)
Motivation	Improving or maintaining functionality for patient and care partner	“Why don’t we start going to the gym…so that we’re not both so…stagnant.” (ID C23) “I take care of myself to be able to take care of him.” (ID C4) “I wanted to make sure that I was…able to help take care of him as well as myself.” (ID C9)
	Successful transplantation	“(The transplant) really motivated him to exercise on a daily basis…We just want to have a better hope and we pray to survive.” (ID C16) “I kind of want to see him on the other side of this…I don’t see him ending his life this way, being on dialysis…it’s not who he is.” (ID C10)
	Enjoyability of exercise	“Every time that [our granddaughter] would come over, we’d go walk around the lake.” (ID C10) “…he works alongside [friends or family] so he can feel normal, like there’s nothing wrong.” (ID C19) “For someone who didn’t want to walk the dog at all…now that’s a guaranteed 3 times a day that he’s out walking for at least 30 minutes each time.” (ID C10)

### Barriers to Pretransplant Exercise and Activity (Patient) or Promoting Pretransplant and Exercise Activity for Their Patient (Care Partner)

#### Capability

A common barrier among patients was a lack of information on an appropriate exercise or activity regimen that would be suitable for patients treated with dialysis (Table 2). As one patient put it, “Who do I go [to]? Nephrologists? They’re busy.” Physical impairment and cognitive impairment were also common barriers, leaving 1 patient feeling like “a dialysis victim for 10 years” and that his body “no longer had stamina.” Several patients reported difficulty remembering to exercise (some because of cognitive impairment), even if counseled, such that 1 patient “couldn’t even do a budget, much less work out.” Furthermore, almost all patients reported symptom-related barriers that could be directly related to dialysis, such as fatigue. Only a minority of patients reported barriers related to their dialysis access (Table S1).

Similarly to patients, care partners also reported that 1 major obstacle to promoting exercise and activity for patients was “a shortage of (exercise-related) information…” leading to a lack of understanding of what would be an appropriate regimen (Table 3).This lack of guidance may in part have been related to gaps in communication with the dialysis or transplantation care teams, resulting in missed opportunities for care partners to intervene, with some care partners saying “it didn’t make me feel like they care about what part I had to take in (the transplantation process)” or that they felt “lost.”

Another common barrier was care partner concerns with their own health, including not just physical impairment but also anxiety and stress. One care partner suggested that the anxiety was especially “taxing, and anything that is emotional will eventually manifest itself physically (in me).” A minority of care partners reported that they already had difficulty with patient adherence even outside of exercise or had tried to promote exercise but had no success due in part to difficulty with getting their patient to focus or be organized (Table S2). No individuals (either patient or care partner) reported weight as a barrier to exercise.

#### Opportunity

Most patients reported difficulty coordinating exercise with their dialysis schedule, with 1 patient describing it as “the feeling of being stuck.” The timing of these interviews was during the height of the coronavirus disease (COVID) pandemic, and therefore some patients who were trying to stay active reported COVID-related disruption in their exercise routines (Table S1).

Many patients required some level of care partner support to be able to exercise. For example, 1 patient who lived on a steep street reported “…to get myself going, I needed [my care partner] to take me and drive me to the park, where it was more level.” These types of environmental or neighborhood-level barriers limited opportunities to exercise for other patients as well (“…if it looks cold outside, I tend to just kind of cower from it).” A small minority of patients reported that nondialysis–related activities interfered with exercise, eg, “I have prioritized making a living over exercise.” (Table S1).

A major theme among care partner responses was lacking enough time to promote patient exercise because of overall burden of time spent, such that with caregiving, bringing in income, and housekeeping they had 3 jobs and that because their patient started dialysis “everything’s gone downhill.” Caregiving was felt to be “rewarding, but…very taxing.” Similarly to patients, the dialysis schedule presented a major opportunity barrier toward exercise, with dialysis described as “all-consuming” and “deflat[ing]…joy in doing things.” Furthermore, for the minority of care partners living away from patients, COVID meant sharply reduced opportunities for interaction (Table S2).

#### Motivation

By far the most common motivational barrier to exercise was dialysis itself, which patients described as “a struggle” and depersonalizing, to the extent that 1 patient described “(existing) every day to… serve as a conduit between (insurance) and the hospitals.” Another patient offered that undergoing dialysis meant that “I didn’t feel like doing anything (else).” Safety concerns (in part related to physical impairment or symptoms associated with dialysis) were another common barrier to motivation, describing “unknown fears” associated with exercise.

Motivational-level barriers to care partners promoting exercise or activity for patients included the perception that “if he doesn’t want to do it, he’s not going to.” Some care partners felt the patient was outright depressed and would tell them they were “tired of dialysis” or that dialysis “would certainly bring my mood down” and therefore they did not feel up to promoting anything other than the necessities of life.

Similarly to patients, care partners also expressed concern about patient safety with activity or exercise, or that they “(worried) about (the patient) going on their own for walks” or felt that they had to serve as “the gatekeeper…(to activity)” to prevent the patient harming themselves inadvertently.

### Facilitators to Pretransplant Exercise and Activity (Patient) or Promoting Pretransplant and Exercise Activity for Their Patient (Care Partner)

#### Capability

The most common facilitator of improving capability for patients to exercise was having a home-based program that would be individualized for the patient (Table 2). For example, one patient reported “I’m not typically a gym person, and I like exercises I can do at home,” and commented further that such a program did not seem to exist for dialysis patients. Another asked specifically for guidance on transitioning from walking to more rigorous home-based exercise. Sub-themes related to this overarching theme were the necessity for external accountability for exercise including feedback on progress (“I need to know that I’m in a system that I can look at results…”). Other patients told us that the presence of structure would also allow for maintenance of exercise: “without [accountability and structure] …as soon as I start feeling better, then I stop.”

About a third of the care partners interviewed suggested that they would feel more capable of promoting exercise for patients if they had support in other ways, including psychosocial support. One care partner told us that “the bottom line is (to hear from their care team), ‘You’re not in this alone.…We’ll figure it out together’” (Table 3).

#### Opportunity

Many patients told us that their care partners actively created opportunities for them to exercise during the day, with a common refrain being that “support from my partner was a big catalyst for getting me going out and pushing myself.” A minority of patients expressed that reopening gyms or other social venues for exercise would give them the opportunity to exercise again (Table S1).

Most care partners were very favorable of joint participation in an exercise program, saying “it would be beneficial for both of us, not just physically, but emotionally.” Care partners also emphasized the importance of individualizing such a program: “Not one size fits all. I don’t believe in that.” Care partners were also in favor of other social support for exercise beyond a health coach or therapist, suggesting that working out with other patients could be beneficial but cautioning that “(patients)’ lives are already expensive.” Finally, most care partners were in favor of trying to incorporate exercise into the daily routine gradually, to avoid over-fatiguing the patient and allow for continued engagement.

#### Motivation

Older patients had a variety of strong motivators for wanting to increase their exercise and activity. Almost all patients reported that they wanted to exercise to improve functionality, eg, “to be able to do things for yourself…feel like I’m not as old,” or that “I just want my body to work again.” This overarching theme included deeply personal goals for many patients, such as spending time with family (“I want to live to see grandkids; “I’ve got to raise my son”), or “to feel like I’m contributing and I have some value.”

Other patients wanted to exercise to improve their success with the transplantation process, such as 1 patient who said “then when you get [transplanted], you don’t have to be in the hospital for a long time.” Or another who told us “If I don’t walk, I don’t get a kidney.”

Another strong motivator outside of the context of health and transplant was having social support for exercise (“when you’re in a group, even if you don’t make your goals, you draw inspiration from others”). A minority of patients expressed motivation to increase activity for weight loss or for activity’s own sake.

Most care partners expressed improving or maintaining functionality for both the patient and themselves as a primary motivator for promoting exercise and activity and “critical for anyone who’s preop, pretransplantation, anything like that.” Furthermore, several care partners expressed that they could not “(help) others if I don’t take care of myself.” Similar to patients, care partners were also motivated to promote exercise in hopes of a successful transplantation. One care partner directly stated that “If you told him, ‘Hey, we need you to increase your activity, we’re about to give you a kidney,’ I’m sure he'll be running around the blocks because that would be his motivation.”

About half of the care partners emphasized that promoting exercise was in part making it enjoyable or “almost…like a game,” with most care partners also expressing that exercise was a route by which they could spend more meaningful time with the patient outside of caregiving, and that “when we feel self-enriched…we can do better jobs.”

## Discussion

The main barriers to an exercise program included the lack of information on appropriate exercise regimens, physical or cognitive impairment (generally related to dialysis), scheduling around dialysis, concerns about safety, and environmental or neighborhood-related barriers. The main motivators and facilitators included a priority of regaining functionality and ability to participate in life, and motivation for successful kidney transplantation. Several barriers (lack of information or guidance, physical impairment, scheduling around dialysis, and concerns about safety) and facilitators (regaining functionality and ability to participate in life, motivation for successful kidney transplant) were shared by the majority of patients and care partners. Furthermore, both patients and care partners reported interest in structured, individualized home-based exercise with accountability and feedback and potentially allowing for joint participation. This study adds to the literature in exercise in dialysis as the first study to our knowledge to describe, from diverse patient and care partner perspectives, barriers, and facilitators to engaging in or promoting pretransplant exercise and activity for older patients awaiting kidney transplantation.

Patients and care partners in this study were of similar age and reported similar relationships compared with other studies in the kidney transplant population.^54^ Patients had similar levels of cognitive dysfunction^55^, ^56^, ^57^ as in other studies of dialysis but a lower prevalence of depression compared with the general dialysis population.^29^^,^^58^ Patients also reported similar barriers to exercise as those elicited in other literature of patients being treated with dialysis.^29^^,^^59^^,^^60^ In particular, both patients and care partners reported significant physical impairment related to dialysis, which aligns with the low SPPB scores we recorded and literature showing that patients treated with dialysis report significantly lower self-reported physical function than the general population,^61^^,^^62^ along with worse quality of life. We were therefore surprised to find that 78% of patients reported consistent engagement with activity (mostly walking). It may be that patients awaiting kidney transplantation are more active than the average older dialysis patient. However, it is important to acknowledge that self-reported activity can differ widely from objective measures of activity,^63^ so it is unclear what threshold of activity (in terms of frequency or intensity) individuals engaged in. Furthermore, because transplantation waitlisting generally requires patients to be below a certain basic metabolic index (<38 at our institution), our study population may have been less likely to report barriers related to weight.

Two studies in particular serve well as comparators to the patient interviews in our study. In a survey of exercise barriers and perceived benefits among younger and older adults treated with dialysis in Canada,^64^ older patients reported improvement of energy and symptoms as primary motivators to exercise in contrast to our study, in which regaining independence was more commonly reported. However, aligning with our findings, survey participants wanted to exercise at home with a combination of aerobic and resistance exercise. Interestingly, in the second study,^65^ providers reported specific systemic barriers that complemented barriers reported by patients in our study, such as difficulty with logistics, concern for safety, and lack of time.

Therefore, it is perhaps not surprising that dialysis itself was the predominant barrier we found in terms of affecting capability to exercise (through physical and cognitive impairment), opportunity to exercise (in terms of schedule), and motivation to exercise. From the care partner standpoint, keeping up with the medical care and complexities of dialysis hampered their ability to focus on exercise. One major systemic barrier of note for both patients and care partners was a lack of readily available information about exercise and activity. Despite the known benefits of exercise in this population^3^^,^^6^ and consensus opinions on the importance of addressing frailty in solid organ transplantation,^66^^,^^67^ nephrologists do not often counsel patients to increase activity.^8^ Furthermore, primary care physicians may feel uncomfortable or unqualified to deliver activity recommendations to this vulnerable population, leading to patients receiving no specific instructions in the crucial pretransplant period. It is notable that 74% of patients and 87% of care partners had never been counseled on exercise. This is likely not helped by significant discrepancies in the guidance regarding suitable modality, frequency, and intensity of activity and exercise in individuals treated with dialysis;^68^ unifying guidance (ideally as a multidisciplinary effort) may lead to better counseling for patients and care partners on benefits and practices of exercise. Care partners in our interviews felt particularly disconnected from the transplantation process. Therefore, to promote active participation in the transplantation process and minimize burden on patients and care partners, exercise guidance may be ideally delivered through incorporating educational sessions into pretransplant evaluation sessions for patient or care partner dyads.

Despite these barriers, both patients and care partners not only reported strong motivation to exercise but also identified a number of important facilitators that could be leveraged to overcome their barriers. For example, many of the reported barriers toward exercise could be overcome using an individualized and structured exercise program involving care partners to support exercise both from a practical sense and for emotional support. Patients and care partners reported more support for home-based than facility-based programs. A home-based program accounting for variations in dialysis schedule and symptomology could significantly improve adherence and engagement, especially if it incorporated exercise or activity suggestions into the patients’ current daily routine.

Patients also reported many motivators toward engagement in physical activity and interventions. The motivator of improving functionality has been highlighted in other qualitative studies of patients treated with dialysis as being important for both patients and care partners.^69^ Previous studies of care partners have focused largely on the enormous burden of care^34^, ^35^, ^36^ that many care partners of patients treated with dialysis report, though a recent qualitative study directly explored the impact of care partners on mobility for patients receiving hemodialysis.^70^ Our study adds further nuance to this complicated issue by directly representing care partner perspectives on barriers and facilitators to promoting exercise and activity for their older patients in the context of prehabilitation before kidney transplantation. Of particular note is the amount of barriers to exercise that patients and care partners share, and their shared interest in joint participation in home-based exercise programs, assuming appropriate structure and guidance are provided.

Based on our findings, and use of the COM-B model, any exercise interventions should consider including the following: care partner participation to promote patient adherence and engagement; structured progression with routine feedback (for example on a weekly or every other week basis); and incorporation of exercise into daily life when possible—including the capacity for home-based exercise (Fig 2). Current guidelines for older adults recommend an additional 150 min/wk of moderately-intense physical activity in addition to 2-3 sessions per of muscle-strengthening exercises,^71^, ^72^, ^73^, ^74^ which may be high for many patients treated with dialysis. However, patients receiving dialysis may benefit from modest increases in activity or exercise even if they are unable to achieve recommended activity levels; some increase in physical activity or exercise is better than none.^22^^,^^62^ Structured progression can be relatively slow—for example, increasing weekly steps by 5% each week, or slowly advancing through home-based exercises by increasing repetitions or resistance (eg, through light weights or resistance bands) and targeting a perceived moderate intensity of exercise (5-7 on a scale of 1-10).

Evidence from the general older population also suggests that participants (especially those with cognitive impairment) have improved trainability and better adherence in exercise interventions if specific measures are taken, including making exercise enjoyable and teaching participants and care partners strategies for goal-setting.^75^ This includes the possibility of joint participation with care partners as above but may also include gamifying exercise through delivering encouraging feedback or other rewards. Care should also be taken to ensure that patient and care partner expectations and goals align before starting any such program. These goals could include goals for physical function set by a health coach or other trained exercise professional, but also self-defined goals that individual participants profess to be motivating for them (eg, spending more time with family). Patients and care partners in our study reported that having external accountability and regular feedback were important facilitators that would promote exercise and physical activity and therefore these should be core features of any such program. A pretransplant exercise program that leveraged social influences (including the care partner or care team) to boost engagement^76^ would align with best practices to make exercise more enjoyable and engaging.^75^ Furthermore, formally incorporating such guidance into the transplant process may help improve exercise even after receiving kidney transplantation because evidence suggests that recipients also require specific exercise guidelines.^77^

Many of our interviewed care partners suggested that such a program may improve their quality of life whether indirectly (through improved patient functioning)^78^ or through joint participation. However, it should be acknowledged that the barriers care partners reported motivating their patients to exercise (eg, health problems, competing priorities for time) could potentially apply to joint participation as well. Therefore, programs incorporating care partner participation and especially joint participation in exercises should be designed to not only use care partner support to promote patient engagement but also to relieve care partner burden where possible.

We acknowledge several important limitations. First, though robust compared with similar studies, our sample size precludes in-depth examination of associations, such as between current exercise status and motivation to continue exercising. Second, our patients were all recruited from the San Francisco or North Bay area and the majority were engaged in walking at baseline (though not necessarily in a structured program or on a regular schedule), meaning these findings may limit generalizability—particularly related to environmental or geographical barriers to exercise. Third, despite the racial and ethnic diversity of our cohort because of the primary language of the interviewers being English, we had limited capacity to interview non-English speakers. In addition, patients and care partners may express different preferences on exercise in the post-COVID environment with facilities reopening.

Finally, we acknowledge that though the inclusion of care partner perspectives is a key strength of this study, the enrollment requirement of patient-care partner dyads may limit applicability of our results to individuals treated with dialysis but lacking a care partner. Such individuals may experience different barriers to exercise and are likely to report different facilitators. Careful consideration should be taken before attempting to apply the results of this study to patients treated with dialysis outside of the context of kidney transplantation.

In conclusion, older patients treated with dialysis and awaiting kidney transplantation reported significant barriers toward exercise in the pretransplant period, as did their care partners. However, participants (both patient and care partner) remained highly motivated to pursue exercise and offered many potential facilitators for future interventions. For example, intervention that include an individualized and structured program of exercise may allow for improvement of physical function and quality of life for both patient and care partner. Further research is needed to determine specifics of such programs including modality and frequency of exercise.
