# Supplementary material for: Barriers and Facilitators to Exercise in Older Adults Awaiting Kidney Transplantation and Their Care Partners
Source: Kidney Med. 2023 Dec 13;6(3):100779. doi: 10.1016/j.xkme.2023.100779 (PMC10900112; doi:10.1016/j.xkme.2023.100779)
Supplement: Supplementary File (PDF) — Tables S1-S2. [file mmc1.pdf]

Table S1. Minor Themes for Patient Barriers and Facilitators to Pre-Transplant Activity and Exercise

COM-B	*Theme*	*Quote*
**Barriers**		
Capability	Dialysis access presenting an issue	" I try to get back to bench pressing again, but they tell you things, but they don't tell you why and then when I keep on asking them, they say "Well, they don't want to affect the vein in my right arm..." So, when I got that explanation, then I stop lifting." (ID P12)<br>"I can't lift too much weights because my arm. So, it's the only arm, one arm hella big and the other arm is going to be hella plinky." (ID P19)<br>"…I cannot move my right arm because of the graft." [P18]
Opportunity	Competing priorities	"I have the cooking. I have to do this. I have to do that, everything. I have to do it on my own and that-- sometimes I get tired." (ID P18)
	COVID limiting ability to exercise	"But since-- with the virus, I'm not taking any chances, you know? I just get in the car and come home." (ID P16)
Motivation	---	---
**Facilitators**		
Capability	---	---
Opportunity	Presence of social venues for exercise	"Well, I had the membership... So, I would say "Hey, I'm spending money." (ID P7)<br>If I could go to the gym…everything is there and I could do it" (ID P12)<br>"You see other people and you kind of compete. You see other people doing it, so it's got to be good." (ID P2)
Motivation	Weight Loss	"If I can increase my physical activity-- I would like to lose weight." (ID P15)<br>"The lower my weight, the, you know, less I have to worry about sugar. And now, I got another thing on top of that, is high blood pressure. The heavier I am, the higher my blood pressure." (ID P11)
	Activity for its own sake	"I think psychologically it's important for me to be active. Walking was a very helpful form of meditation really." (ID P6)

	“I just ran, ran like I was running because that was my thing, running. So I can't go far but around the neighborhood, might do a little slow motion. I used to love to run.” (ID P4)<br>“When you are exercising, your body [feels] better and it keeps your mental state clear” (ID P7)

Table S2. Minor Themes for Care Partner Barriers to Promotion of Pre-Transplant Activity and Exercise for Patients

COM-B	*Theme*	*Quote*
**Barriers**		
Capability	Patient adherence already difficult	“Focusing, paying attention to instructions, that is the key thing, [they need constant] instruction.” [ID C14]
Opportunity	COVID as a barrier	“It becomes much more of a challenge. I can’t even imagine how much of a challenge it is for other patients who relied on things like physical therapy and group classes and all that.” (ID C21)
Motivation	---	---
